# Structural and Functional Investigation of Flavin Binding Center of the NqrC Subunit of Sodium-Translocating NADH:Quinone Oxidoreductase from *Vibrio harveyi*

**DOI:** 10.1371/journal.pone.0118548

**Published:** 2015-03-03

**Authors:** Valentin Borshchevskiy, Ekaterina Round, Yulia Bertsova, Vitaly Polovinkin, Ivan Gushchin, Andrii Ishchenko, Kirill Kovalev, Alexey Mishin, Galina Kachalova, Alexander Popov, Alexander Bogachev, Valentin Gordeliy

**Affiliations:** 1 Moscow Institute of Physics and Technology, Dolgoprudniy, Russia; 2 Institute of Complex Systems (ICS-6) Structural Biochemistry, Research Centre Jülich GmbH, Jülich, Germany; 3 Belozersky Institute of Physico-Chemical Biology, Lomonosov Moscow State University, Moscow, Russia; 4 A.N. Bach Institute of Biochemistry, Russian Academy of Sciences, Moscow, Russia; 5 European Synchrotron Radiation Facility, Grenoble, France; 6 Univ. Grenoble Alpes, IBS, Grenoble, France; 7 CNRS, IBS, Grenoble, France; 8 CEA, IBS, Grenoble, France; University of South Florida College of Medicine, UNITED STATES

## Abstract

Na^+^-translocating NADH:quinone oxidoreductase (NQR) is a redox-driven sodium pump operating in the respiratory chain of various bacteria, including pathogenic species. The enzyme has a unique set of redox active prosthetic groups, which includes two covalently bound flavin mononucleotide (FMN) residues attached to threonine residues in subunits NqrB and NqrC. The reason of FMN covalent bonding in the subunits has not been established yet. In the current work, binding of free FMN to the apo-form of NqrC from *Vibrio harveyi* was studied showing very low affinity of NqrC to FMN in the absence of its covalent bonding. To study structural aspects of flavin binding in NqrC, its holo-form was crystallized and its 3D structure was solved at 1.56 Å resolution. It was found that the isoalloxazine moiety of the FMN residue is buried in a hydrophobic cavity and that its pyrimidine ring is squeezed between hydrophobic amino acid residues while its benzene ring is extended from the protein surroundings. This structure of the flavin-binding pocket appears to provide flexibility of the benzene ring, which can help the FMN residue to take the bended conformation and thus to stabilize the one-electron reduced form of the prosthetic group. These properties may also lead to relatively weak noncovalent binding of the flavin. This fact along with periplasmic location of the FMN-binding domains in the vast majority of NqrC-like proteins may explain the necessity of the covalent bonding of this prosthetic group to prevent its loss to the external medium.

## Introduction

Na^+^-translocating NADH:quinone oxidoreductase (NQR) is a redox-driven sodium pump that generates a transmembrane difference in electrochemical Na^+^-potential [1]. This enzyme has been shown to operate in the respiratory chain of various bacteria, including such pathogenic microorganisms as *Vibrio cholerae*, *Klebsiella pneumoniae*, *Haemophilus influenzae*, *Neisseria gonorrhoeae*, *Neisseria meningitidis*, *Yersinia pestis*, *Pseudomonas aeruginosa*, *Porphyromonas gingivalis*, and many others [2]. NQR consists of six subunits (NqrA–F) encoded by six genes of the *nqr* operon [3, 4] and has a unique amino acid sequence and set of prosthetic groups [1]. The enzyme contains a [2Fe-2S] cluster, a FeS center, noncovalently bound flavin adenine dinucleotide (FAD) and riboflavin, as well as two covalently bound FMN residues [5]. The latter two prosthetic groups are attached to threonine residues in subunits NqrB and NqrC [6]. Flavinylation of these subunits is catalyzed by a Mg^2+^-dependent flavin transferase (ApbE) using FAD as substrate [7]. This type of covalent bonding of the flavin is rare and can be found only in NQR subunits and related bacterial proteins such as RnfD and RnfG subunits of sodium-dependent NADH:ferredoxin oxidoreductase (so called RNF complex) [8], regulator of NO reductase transcription (NosR) [9], soluble cytoplasmic fumarate reductase (KPK_2907) [10], and urocanate reductase (UrdA) [11]. Such unusual type of flavin covalent bonding implies an atypical structure of the flavin-binding pocket. However, there were no data on proteins containing covalently bound FMN residue in structural databases. In spite of 3D structures of four different NqrC-like proteins having been solved (PDB: 3LWX, 3DCZ, 2KZX, and 3O6U), all these structures were determined for flavin-free, apo-forms of the corresponding proteins. Just recently, a 3D structure of holoNqrC from *V*. *cholerae* has been reported [5]. The reason of FMN covalent binding in NqrC is not currently clear. Therefore, to answer this question, we developed a method for production of apo- and holo-forms of the water soluble flavin-binding domain of the NqrC subunit (NqrC') based on heterologous expression of the *nqr*C' gene in *Escherichia coli* cells in the absence or in the presence of co-expressed gene of flavin transferase, respectively [7]. In the present work, flavin binding properties of apo- and holo-forms of *V*. *harveyi* NqrC' were investigated by measuring of apoNqrC' affinity to FMN and structural analysis of the holo-form of this protein.

## Results

### Noncovalent binding of free FMN to apoNqrC′

**Fig 1:**
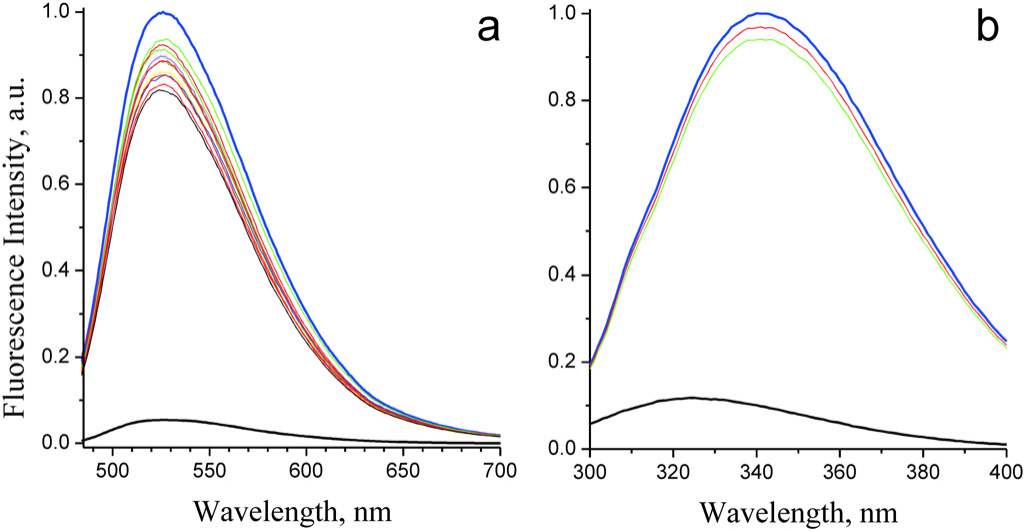
Noncovalent binding of free FMN to apoNqrC' studied by the flavin and the protein fluorescence shown on the left and right panels, respectively. (a) Fluorescence of 0.35 μM free FMN (thick blue line) and 0.35 μM holoNqrC' (thick black line). The thin lines represent titration of fluorescence of free FMN (0.35 μM) by increasing amounts of apoNqrC' (apoNqrC' concentrations varied from 0.08 to 45 μM). (b) Tryptophan fluorescence of 0.32 μM apoNqrC' (thick blue line) and 0.32 μM holoNqrC' (thick black line). The red and green thin lines represent fluorescence of 0.32 μM apoNqrC' in the presence of 0.32 and 0.64 μM of free FMN, respectively.

The flavin-free apo-form of NqrC' and the holo-form of this protein containing a covalently bound FMN residue were produced in *E*. *coli* cells both in the absence and in the presence of co-expressed gene of flavin transferase, respectively [7]. Free FMN exhibited a high fluorescence around 525 nm. As shown on Fig. 1a, covalent binding of FMN residue in holoNqrC' resulted in almost complete quenching of the flavin fluorescence. This effect can be used for determination of noncovalent binding of FMN to apoNqrC'. To accomplish this, free FMN was titrated with varying amounts of apoNqrC'. However, this titration did not quench the fluorescence of the flavin (Fig. 1a). Even high concentrations of apoNqrC' above 45 μM (more then 100-fold excess of the apoprotein comparably to free FMN) proved to be ineffective indicating only weak noncovalent binding of free FMN to apoNqrC'.

The apoNqrC' fluorescence showed a maximum at 342 nm with excitation wavelength of 280 nm (Fig. 1b), presumably due to fluorescence of a tryptophan residue(s). This fluorescence was quenched in holoNqrC' with concomitant shift of the emission maximum to 325 nm. Addition of free FMN to apoNqrC' solution did not result in quenching of the protein fluorescence (Fig. 1b). These data support the conclusion that apoNqrC' is unable to form proper FMN-protein complex without covalent bonding of the prosthetic group.

It cannot be excluded that properties of [NqrC'–noncovalently bound FMN] complex differ from those of holoNqrC' with covalently bound FMN, and only covalent bonding of FMN residue is capable to provide a strong quenching of protein and flavin fluorescence. To test this possibility, the noncovalent binding of free FMN to apoNqrC' was also studied by isothermal titration calorimetry. However, mixing of apoNqrC' with FMN did not result in any significant heat changes. The observed effect was <<0.1 kcal/mol of FMN (data not shown), which is more than 100-fold lower comparing to FMN binding by flavoproteins [12, 13]. Thus, affinity of apoNqrC' to free FMN is very low, and formation of a phosphoester bond between Thr229 and FMN residues is essential for further binding of the isoalloxazine moiety of the flavin.

To study structural aspects of flavin binding to NqrC, the holo-form of NqrC' was crystallized and its 3D structure was solved at 1.56 Å resolution.

### Crystal packing

The crystal structure of the holoNqrC' protein with a covalently bound FMN residue was determined and refined at 1.56 Å resolution. The final model includes protein molecule (residues 36–261, residues are enumerated in accordance with full-length NqrC UniProt number: M7R347) and 114 water molecules in one asymmetric unit. No electron density was observed for residues 33–35 although they were verified to be present in the expressed construct. It is probably related to the structural flexibility of terminal residues.

**Fig 2:**
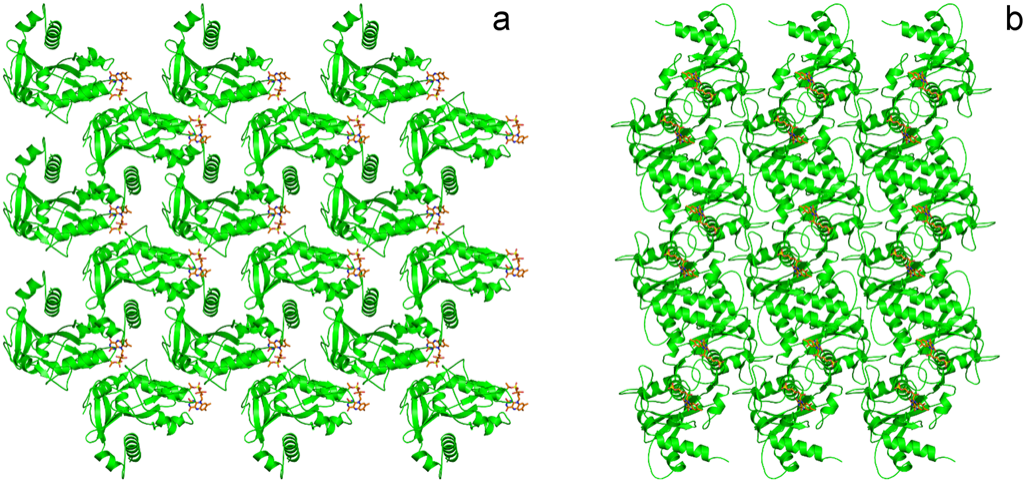
Crystal packing of the holoNqrC' protein. FMN residue is shown in orange. (a) and (b)—along *a* and *c* axis, respectively.

Fig. 2 shows the packing of the holoNqrC' protein in the crystal. The protein was crystallized in P2_1_ symmetry in agreement with recently reported data [5, 14]. However, cell parameters are different between in the two studies (a = 39.6 Å, b = 55.4 Å, c = 46.6 Å, β = 91.7° in present study and a = 46.7Å, b = 41.7 Å, c = 61.4 Å, β = 107.7° in [14]). It results in different solvent content (33.7% and 47.5% correspondingly) which correlates with better resolution (1.56 Å) obtained in present study (compared with 1.7 Å in [5, 14]).

Crystal contacts (excluding those mediated by water molecules) are formed by 13 residues, all of which either located in loops (Glu58 and Gly59 in loop A-B; Lys78 in loop1–2; Glu84 in loop 2-C; Lys102 in loop C-D; Asp113 and Glu114 in loop 3–4; Gly148 in loop 6–7; Lys197 and Lys204 in loop F-9; Lys211 in loop 9–10) or are terminal residues of minor secondary structure elements (Pro104 of 3_10_-helix D and Asp223 in β-strand 10). The only exception is the flavinylated Thr229 at the beginning of extensive α-helix G. The FMN residue strongly participates in the crystal stabilization forming direct contacts with Lys109 and Glu114 ($O_{1P}^{FMN}-N_{}^{Lys109}$;$O_{3\prime }^{FMN}-O_{E1}^{Glu114}$;$O_{3\prime }^{FMN}-O_{E2}^{Glu114}$;$O_{2\prime }^{FMN}-O_{E2}^{Glu114}$) and mediated by water molecules contacts with Ser107, Lys109 and Arg121 ($O_{1P}^{FMN}-W66-O_{}^{Ser107}$, $O_{3\prime }^{FMN}-W50-O_{}^{Lys109}$, $O_{1P}^{FMN}-W66-N_{H2}^{Arg121}$,$O_{2P}^{FMN}-W143-W50-O_{}^{Lys109}$).

### General features of holoNqrC′ structure

**Fig 3:**
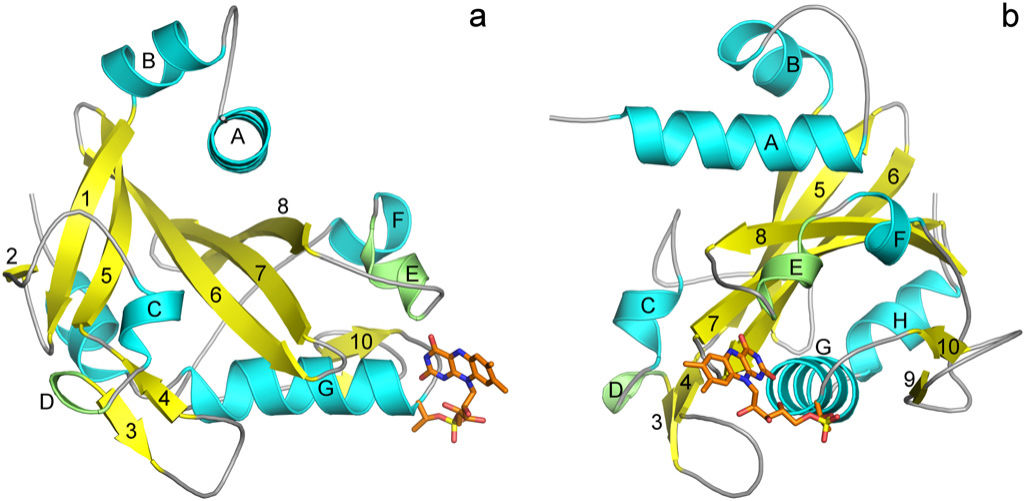
HoloNqrC' structure. (a) and (b)—Overall view with 90°-rotation. Different secondary structure elements are shown in colors: β-sheets in yellow, α-helices in cyan, 3_10_-helix in green. β-strands and helices are designated with numbers and Latin latters, respectively. Secondary structure was assigned with DSSP [29].

The structure of holoNqrC’ from *V*. *harveyi* is similar to recently published 4U9S [5] (an analog from *V*. *cholera*) with the RMS for Cα equals 0.434 Å. The central feature of the holoNqrC' protein is a twisted antiparallel β-sheet formed by six β-strands: 1, 2, 5–8 (Fig. 3). The β-sheet is surrounded by α-helices from both sides (the helix A from one side and the helices G and H from the other), which fit to the grooves of the β-sheet. The loops between described secondary structure elements also form regular but less extensive structures. The loop between α-helix A and β-strand 1 forms α-helix B. An extensive region between β-strands 2 and 5 (both participating in the central β-sheet) contains one short (5 residues) α-helix C, 3_10_-turn D and a short (3 residues in each strand) antiparallel β-sheet 3–4. Another extensive region between β-strand 8 and α-helix G contains two short helices: 3_10_-helix E and α-helix F (5 residues each); and a short (3 residues in each strand) parallel β-sheet 9–10. The FMN residue is covalently attached to Thr229 at the beginning of α-helix G.

### Structure similarity search

The search in PFAM database (http://pfam.xfam.org/) shows that holoNqrC' belongs to the FMN binding family (PFAM ID accession code PF04205) with a high E-Value of 2.6e^-16^ and bit score of 59.5. Interestingly, this family does not belong to 23 structural clans described for flavin-dependent proteins [15]. Besides structures of NqrC' from *V*. *harveyi* (current work) and *V*. *cholerae* [5] there are only 4 PDB structures of proteins from PF04205 family (3LWX, 3DCZ, 2KZX, and 3O6U). Two of them (2KZX and 3O6U) are uncharacterized proteins from *Clostridium thermocellum* and *Clostridium perfringens*, respectively; 3DCZ is a putative RnfG subunit of electron transport complex from *Thermotoga maritima*. The last one (3LWX) is the flavin-binding domain of the NqrC subunit from *Parabacteroides distasonis*. This structure was used as a model in the present study for molecular replacement. 3LWX is similar to the structure obtained here with RMS for Cα of 1.077 Å. It is noteworthy that all four of these structures were obtained for the apo-forms of the corresponding proteins, which do not contain a covalently bound flavin.

Subsequent 3D-alignment of holoNqrC' with known structures was done using DALI [16] (http://ekhidna.biocenter.helsinki.fi/dali_server) and deconstruct (http://epsf.bmad.bii.a-star.edu.sg/struct_server.html) servers which both gave similar results. Two members of “FMN binding” family (3LWX and 3DCZ) are present at top two positions in both similarity lists (DALI Z-score 21.6 and 8.6, respectively). Surprisingly, the two other members of “FMN binding” family (2KZX and 3O6U) are present in summary list with considerably lower Z score (DALI Z-score 2.7 and 3.8, respectively). This contradiction between 3D and sequence alignments can be solved if we describe minimal scaffold of the FMN-binding domain as the β6–β8-sheet followed by the G and H α-helices. 3LWX shows a low value of rmsd (2.5 Å for backbone atoms) and significant number of structurally equivalent residues (95% of total number of residues) with NrqC' of present study, which explains the high DALI Z-score (21.6). The situation is somewhat worse for 3DCZ (where 3.1 Å rmsd was obtained for 75% of structurally equivalent residues) resulting in lower DALI Z-score (8.6). Finally, 2KZX and 3O6U have rmsd of 2.9 Å and 3.1 Å with 67% and 61% of structurally equivalent residues, correspondingly. Only residues from region 142–246 were found to be structurally equivalent to regions of 2KZX and 3O6U with significant deviations for all other parts of the proteins. This fact may explain why proteins from the same family show so low DALI Z-scores (2.7 and 3.8, comparing 3DCZ to 2KZX and 3O6U, respectively). Notably, the structurally equivalent region corresponds well to the earlier predicted FMN-binding domain [17] with a good match of the predicted secondary structure elements.

### FMN conformation and flavin binding site

**Fig 4:**
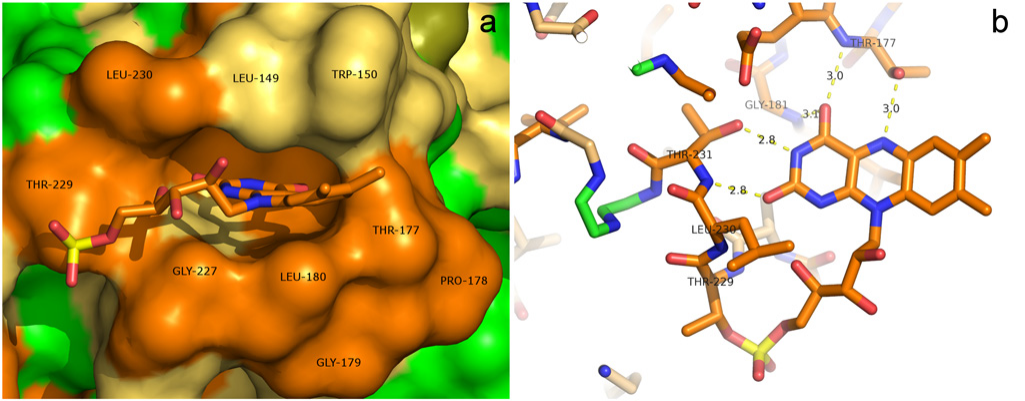
FMN binding site of holoNqrC'. The protein is shown as a space-filling model at (a). H-bonds stabilizing the conformation of FMN residue are shown at (b). The intensity of orange color represents the 4 levels of amino acid conservation in agreement with Fig. 5. Green color represents nonconservative amino acids.

The NqrC' was subjected to flavinylation by flavin transferase ApbE *in vivo* prior to the crystallization resulting in one FMN-molecule bound per protein molecule via Thr229 [7]. The electron density of Thr229 side chain unambiguously demonstrates its flavinylation. The FMN binding pocket of holoNqrC' (shown on Fig. 4) is formed by N-termini of α-helix G and 3_10_-helix E, as well as by loops 6–7, 8-E and 10-G. The cavity is inlayed by amino acids conservative among NqrC subunits from different bacteria (shown in orange on Fig. 4a): Leu149, Trp150, Glu176, Thr177, Leu180, Gly181, Gly227, Ala228, Leu230 and Thr231. The N-terminus of α-helix G together with the next following loop 10-G (residues 227–231) harbors the flavinylated Thr229 and creates a groove for the ribosyl part of FMN. The position of the FMN-phosphate along with its covalent bond to Thr229 is stabilized by an H-bond interaction with backbone of Gly227. The interaction is mediated by water molecule ($O_{2P}^{FMN}-W97-N_{}^{Gly227}$) and by an H-bond with the backbone of Lys109 of the adjacent symmetry-related protein molecule ($O_{1P}^{FMN}-N_{}^{Lys109}$). The isoalloxazine ring of FMN is squeezed between two loops (6–7 and 8-E with the most involved Leu149 and Leu180) and flanked by α-helix G and 3_10_-helix E. The position of the ring is stabilized by H-bonds with Thr177 and Thr231 side chains ($N_{5}^{FMN}-O_{G1}^{Thr177}$,$N_{3}^{FMN}-O_{G1}^{Thr231}$) as well as with backbone of Gly181 and Thr177 ($O_{4}^{FMN}-N_{}^{Thr177}$,$O_{4}^{FMN}-N_{}^{Gly181}$). Surprisingly, the position of Trp150 suggests it should stack with the isoalloxazine ring but its conformation happened to be not suitable for this interaction. It is possible that this may be an artifact caused by the crystal contacts and *in vivo* conformation of Trp150 may differ from that in a crystal.

**Fig 5:**
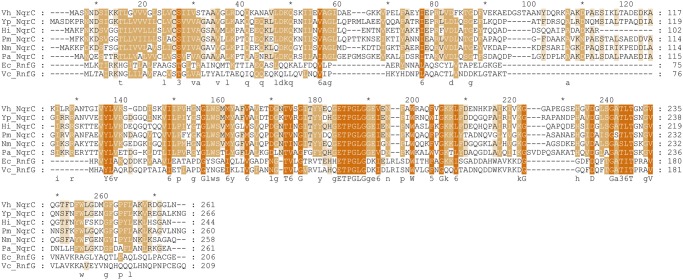
Sequence alignment of the NqrC subunits of NQR from different bacteria (*V*. *harveyi* (*Vh*_NqrC), *Yersinia pestis* (*Yp*_NqrC), *Haemophilus influenza* (*Hi*_NqrC), *Pasteurella multocida* (*Pm*_NqrC), *Neisseria meningitidis* (*Nm*_NqrC) and *Pseudomonas aeroginosa* (*Pa*_NqrC)) as well as paralogous RnfG subunits of the RNF complex from *E*. *coli* (*Ec*_RnfG) and *V*. *cholerae* (*Vc*_RnfG). The intensity of orange color represents the 4 levels of amino acid conservation (calculated in [30]).

Sequence alignment of the NqrC proteins and the paralogous RnfG proteins is shown in Fig. 5. Notably, the region of Asp223-Thr229 is highly conserved. A probable reason is that this region forms the flavinylation motif for FMN attachment to Thr229 by flavin transferase [7]. It is more striking that Glu176-Gly181 region (ETPGLG motif) and Lys211 are absolutely conserved. Our structure (Fig. 4) explains conservation of these amino acids: all mentioned above residues belong to flavin binding pocket and are involved in its noncovalent binding. High conservation of the amino acids means that flavin binding is highly specific and this is necessary for NqrC to fulfill its function.

**Fig 6:**
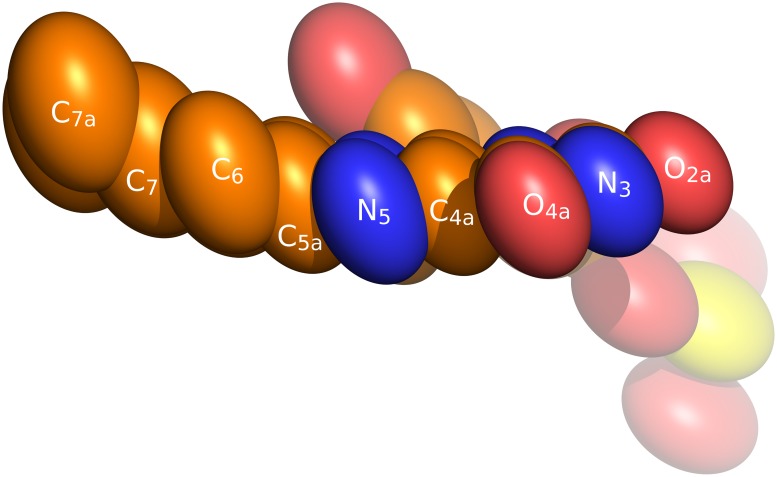
Ellipsoids of atomic-displacement parameters of the FMN residue in NqrC' drawn at 50% probability. Numeration of the isoalloxazine atoms is shown according to the IUPAC nomenclature.

As can be seen in Fig. 4, the FMN adopts a “butterfly”-conformation with ~20° angle between the planes formed by the pyrimidine and benzene rings. This conformation of the isoalloxazine ring is unusual for the oxidized form (which was used for crystallization). Detailed examination of the experimental atomic displacement parameters of the FMN atoms (shown on Fig. 6) shows that the benzene part of the isoalloxazine moiety exhibit disorder perpendicular to the molecular plane. It was proposed previously [18] that this flexibility (as well as “butterfly”-conformation itself) may be a record of structural distortion and partial flavin reduction accumulated during X-ray data collection. The conformation of FMN in NqrC most probably corresponds to the intermediate X-ray-radiation-induced one-electron-reduced state of FMN as was proposed in the abovementioned reference.

In constrast to the present structure, FMN in 4U9S [5] adopts a planar conformation. Most probably, this discrepancy can be accounted for by different redox states of FMN residues in these structures, namely the oxidized form of this prosthetic group in 4U9S and the radical form in the present structure.

## Discussion

The presence of covalently bound flavin in the NqrC subunit of NQR was firstly observed by Zhou et al. [19]. Later Hayashi et al. showed that FMN is covalently bound to Thr229 of this subunit [6]. Based on the MALDI-TOF mass spectrum of a fluorescent peptide derived from NqrC as well as on the spectrum of its fragmentation products, it was proposed that the covalently bound FMN residue is attached to Thr229 via a phosphoester bond [6]. In the present work, we confirm that directly by a crystallographic analysis of holoNqrC'. Analysis of F_o_—F_c_-maps shows clear electron density for the atoms comprising the FMN residue as well as the phosphoester bond, which covalently links FMN to Thr229 that is in a full accordance with earlier prediction [6]. The unusual type of covalent attachment of the flavin in NqrC-like proteins may be caused by an atypical structure of the corresponding flavin-binding domains. Accordingly, the NqrC' fold determined in the present work does not belong to 23 structural clans described for flavoproteins [15] thus representing a new class of FMN-binding proteins.

The NqrC subunit plays a role of an electron carrier within NQR. Under physiological conditions, this subunit can transfer only one electron [20], which is provided by stabilization of the anionic semiquinone form of the FMN residue in this subunit [21, 22]. This redox property is retained in the truncated form of NqrC used in the current study [7]. The obtained electron density of the holoNqrC' protein in the crystals clearly shows that the isoalloxazine moiety of the FMN residue adopts a bended “butterfly”-conformation with ~20° angle between the planes formed by the pyrimidine and benzene rings. This conformation of FMN most likely corresponds to the radiation-induced one-electron-reduced (semiquinone) state of the flavin [18], which is in accordance with relatively high stability constant for anionic radical of the FMN residue in NqrC [22]. This observation can illustrate structural aspects of NqrC followed from the mentioned above redox properties of the FMN residue. As shown in the current work, the isoalloxazine moiety of the FMN residue is buried in a hydrophobic cavity in such manner that its pyrimidine ring is squeezed between Leu149 and Leu180 while its benzene ring is extended from the protein surroundings. The latter fact is expected to allow flexibility of the benzene ring, which can help the FMN residue to take the bended conformation at one-electron reduction.

The properties of the flavin-binding pocket in NqrC (described above) which resulted in significant flexibility of its isoalloxazine ring may also lead to relatively weak noncovalent binding of the flavin to protein. Flexibility can also explain the low affinity of free FMN to apoNqrC', determined in the current work. On the other hand, analysis of bacterial proteins containing a covalently bound FMN residue reveals that they are periplasmic proteins in most cases [10]. Thus, weak noncovalent binding of the flavin along with periplasmic location of the FMN-binding domain may explain the necessity of the covalent bonding of this prosthetic group to prevent its loss to the external medium.

## Materials and Methods

### Isolation of recombinant 6×His-tagged NqrC′ protein

The preparations of the truncated NqrC subunit, NqrC' (a soluble variant of NqrC without its N-terminal transmembrane α-helix, containing 33–261 amino acid residues of the full length NqrC and a C-terminal 6×His tag), were obtained as described previously [7]. The apoNqrC' protein was purified from *E*. *coli* cells bearing the pMshC3 plasmid. The holoNqrC’ protein was produced in *E*. *coli* bearing both pMshC3 and pδhis3 plasmids. For *nqr*C' or *nqr*C'+*apb*E' induction, *E*. *coli* cells were grown at 32°C to mid-exponential phase (*A*_600_ = 0.3–0.4)The growth medium was supplemented with 0.2% (w/v) L-arabinose and cells were grown for an additional 3.5 h. The cells were harvested by centrifugation (10,000*g*, 10 min) and washed twice with medium containing 300 mM NaCl, 10 mM Tris-HCl, and 5 mM MgSO_4_ (pH 8.0). The pellet was suspended in medium containing 300 mM NaCl, 20 mM Tris-HCl, 5 mM MgSO_4_, 1 mM phenylmethylsulfonyl fluoride, and 5 mM imidazole-HCl (pH 8.0), and the suspension was passed twice through a French press (16,000 psi). Cell debris and membrane vesicles were removed by centrifugation at 180,000*g* (60 min).

6×His-tagged apoNqrC' or holoNqrC' were purified from the appropriate supernatant using affinity chromatography. This was accomplished by loading a supernatant onto a Ni-NTA column equilibrated with solution A containing 300 mM NaCl, 10 mM Tris-HCl, and 5 mM imidazole-HCl (pH 8.0); washing the column twice, first with solution A containing 10 mM imidazole-HCl and then with solution A containing 20 mM imidazole-HCl; and eluting apoNqrC' or holoNqrC' with solution A containing 100 mM imidazole-HCl. The protein obtained was then concentrated, supplemented with the Halt protease inhibitor cocktail, and kept frozen at -80°C until use. Protein concentration was determined by the bicinchoninic acid method [23] using bovine serum albumin as a standard. Concentrations of holoNqrC' were also determined using extinction coefficient *ε*_452–600_ = 13.4 mM^-1^ cm^-1^ [7].

### Determination of noncovalent binding of free FMN to apoNqrC′ using the flavin and the protein fluorescence

Noncovalent binding of FMN to apoNqrC' was measured by quenching of FMN fluorescence by apoNqrC'. Solution of free FMN at 0.35 μM in 50 mM potassium phosphate buffer (pH = 7.0) was titrated with varying concentrations of apoNqrC' (from 0.08 to 45 μM). Fluorescence emission spectra were recorded at room temperature with a FluoroMax-3 spectrofluorometer (Horiba Jobin Yvon) using an excitation wavelength of 450 nm. To estimate fluorescence quenching at 100% FMN binding, emission spectrum of 0.35 μM holoNqrC' was determined under the same conditions.

Noncovalent binding of FMN to apoNqrC' was also determined by quenching of apoNqrC' tryptophan fluorescence by free FMN. The apoprotein at 0.32 μM in 50 mM potassium phosphate buffer (pH = 7.0) was titrated with varying amounts of FMN. Fluorescence emission spectra were recorded at room temperature with a FluoroMax-3 spectrofluorometer using an excitation wavelength of 280 nm. To estimate the protein fluorescence quenching at 100% FMN binding, emission spectrum of 0.32 μM holoNqrC' was determined at the same conditions.

### Determination of noncovalent binding of free FMN to apoNqrC′ by means of isothermal titration calorimetry

Thermodynamic measurements of FMN binding to apoNqrC' were made using an isothermal titration calorimeter VP-ITC (MicroCal Ltd.) with a 1.4-ml cell at 12°C. Before experiments, apoNqrC' was dialyzed against 50 mM potassium phosphate buffer (pH = 7.0). All samples were degassed before the experiment. Reference titrations injecting FMN to the buffer were carried out and the results were used as a reference for the FMN–apoNqrC' titrations. A typical injection schedule included the addition of 15 μl samples of 0.5 mM FMN to 50 μM apoNqrC' with 30 injections at three minute intervals. For the first injection only 5 μl of FMN was added and the corresponding data point was deleted from the analysis. Data analysis was carried out using MicroCal Origin 7.0 software with the “one set of sites” model.

### Crystallization

HoloNqrC′ was concentrated to a final concentration of 60 mg/ml and crystallized at 22°C by the sitting-drop vapor-diffusion method using a nanoliter-volume liquid handler NT8 (Formulatrix). Crystallization drops were set up by mixing 100 nl protein solution with 100 nl of a reservoir solution consisting of 0.1 M K thiocyanate, 20–30%(w/v) PEG MME 2000, pH 6.8 against 50 μl of reservoir solution. Large thin plate-like yellow crystals grew upto 400 μm×200 μm×20 μm size. Crystals grew very slowly (~ half a year) and usually not more then 1–3 crystals were found in any single crystallization probe. Crystals had strong tendency to be twined. The crystals were mounted in nylon loops (Hampton Research) and flash cooled in liquid nitrogen without any further cryoprotector added.

### X-ray data collection

X-ray diffraction data were collected at the beamline ID29 of the ESRF at 100 K (wavelength 0.976 Å) equipped with PILATUS 6M detector. Several other supportive data sets were collected at ID23–1.

### Data treatment

X-ray data were integrated and scaled in XDS [24] at 1.56 Å resolution. Crystallographic statistics is given in Table 1. Initial phases were determined by molecular replacement similar to the procedure described in [14] using the structure of NqrC from *Parabacteroides distasonis* (PDB-code: 3LWX). Initial model was placed with MOLREP [25] and further refined with *phenix*.*rosetta_refine* [26]. Automated model building as well as further model refinement was done in PHENIX [27]. Manual model refinement was done in COOT [28]. The final statistic of the model is given in Table 1.

**Table 1:** Crystallographic table.

**Data collection**	
Space group	P2_1_
Cell dimensions	
*a*, *b*, *c* (Å)	39.6 55.4 46.6
α, β, γ (°)	90 91.7 90
Resolution (Å)	46.53–1.56 (1.60–1.56)
*R*_merge_ (%)	7.4 (162.3)
*I*/*σI)*	9.6 (0.6)
Completeness (%)	96.6 (74.2)
CC(1/2)	99.9 (10.8)
**Refinement**	
Resolution (Å)	46.53–1.56
No. reflections	27689
*R*_work_ / *R*_free,_(%)	19.9 / 22.2
Number of atoms	1908
R.m.s. deviations	
Bond lengths (Å)	0.005
Bond angles (°)	0.944

### Accession numbers

Atomic coordinates and structure factors have been deposited in the Protein Data Bank with accession code 4XA7.
